# Cytogenetic Study on the Biostimulation Potential of the Aqueous Fruit Extract of *Hippophae rhamnoides* for a Sustainable Agricultural Ecosystem

**DOI:** 10.3390/plants9070843

**Published:** 2020-07-04

**Authors:** Elena Bonciu, Aurel Liviu Olaru, Elena Rosculete, Catalin Aurelian Rosculete

**Affiliations:** 1 Department of Agricultural and Forestry Technology, Faculty of Agronomy, University of Craiova, 13 A.I. Cuza Street, 200585 Craiova, Romania; elena.bonciu@edu.ucv.ro (E.B.); catalin.rosculete@edu.ucv.ro (C.A.R.); 2 Department of Land Measurement, Management, Mechanization, Faculty of Agronomy, University of Craiova, 13 A.I. Cuza Street, 200585 Craiova, Romania

**Keywords:** seabuckthorn, *A. cepa*, AESF, growth, cytotoxic, genotoxic, environment

## Abstract

This cytogenetic study evaluates the biostimulation potential of the aqueous extract of seabuckthorn fruits (AESF) in plant cells, using the *Allium cepa* species as a test plant. The effects were monitored both at the macroscopic and microscopically level. The onion bulbs were exposed to the action of different concentrations of AESF (0.5, 1, 1.5, 2, and 2.5%) for 72 h. The obtained results showed the positive effect induced by the aqueous extract on the growth of the meristematic roots, but only at concentrations ranging between 0.5–1.5%, when the average length of the roots had values between 2.51–3.40 cm, which means an increase compared to the untreated control with 3.71–40.49%. Within the same concentration range of the AESF, an effect of intensifying the mitotic activity was recorded. On the other hand, at the 2–2.5% concentration of the AESF, there was an inhibitory effect on the growth of meristematic roots. Additionally, concentrations ≥2% of AESF induced a cytotoxic and genotoxic effect through the occurrence of some chromosomal and nuclear abnormalities in *A. cepa* cells (sticky, laggards, ring chromosomes, and micronucleus). The obtained results suggest the biostimulation potential of the AESF for plant cells and the possibility of using it as an eco-friendly fertilizer.

## 1. Introduction

Natural ecosystems offer a variety of plants with multiple benefits, both for the health of the human body, and for the food, pharmaceutical industry, etc. Natural phytochemicals are provided by diverse intrinsic rich sources such as fruits, leaves, branches, as well as roots of different plants [1]. Of these plants, seabuckthorn is considered to be a wonder plant due to its rich and extremely varied content of bioactive phytochemical compounds [2,3,4]. The white seabuckthorn (*Hippophae rhamnoides*) is one of the spontaneous shrubs of special value for the food, pharmaceutical, cosmetic, etc., due to its rich content in bioactive phytochemicals. The content in vitamin C exceeds twice that of blueberries and about 10 times that of citrus fruits, and the amount of vitamin C is higher in well ripened fruits, reaching up to 400–800 mg/100 g of juice [3]. White seabuckthorn is also rich in other vitamins (A, B1, B2, B6, E, F, K, P) and is often called a natural polyvitamine [2,5].

Research has shown that seabuckthorn fruits contain a number of valuable biologically active substances such as β—carotene, organic acids, essential oils, polyphenols, flavonoids, phytosterols, tocopherols, vitamins, polyunsaturated fatty acids, coumarins, triterpens, protein (globulins, albumins), amino acids, carbohydrates, minerals [6,7]. These active compounds play a very important role in regulating the human metabolism, with therapeutic and curative action in the prevention and treatment of eye and skin diseases, juvenile acne, gastroenteritis, chronic hepatitis, kidney impairment, high blood pressure, avitaminosis, diseases of the nervous system, burns, etc. [4,8,9]. Seabuckthorn oil contains 10 times more carotene than carrot, it has an anti-bacterial, sedative action, accelerating tissue recovery [3]. Negi et al. (2005) show that methanolic extracts from seabuckthorn inhibit the occurrence of oxygen free radicals and lead to the removal of existing radicals [9].

Seabuckthorn fruits are the ones mainly used, but to enhance the health for consumers, the pulp remaining after extracting the juice, seeds, leaves, etc. is also of great importance [10]. Due to all these benefits, seabuckthorn can be used as a general toning agent for the human body [3].

Through the color contrast between leaves (white-silver) and fruit (yellow-orange, remaining on the plant also during winter), seabuckthorn is used in landscape architecture as an ornamental plant [3]. Due to its high scouring capacity, seabuckthorn contributes considerably to the sustainable restoration of highly degraded lands, by quickly fixing and consolidating them [11]. Additionally, seabuckthorn is important for improving soil characteristics due to its ability to assimilate atmospheric nitrogen directly through symbiotic bacteria located in roots [12].

Agriculture is a vital activity, on which food security and the balance of ecosystems rely. The sustainable agricultural ecosystem involves obtaining safe and constant yields, with minimal negative effects on the environment. Agriculture is a source of economic development and livelihood, but pollution can lead to a number of environmental and health hazards and induce alteration of crop yield quantity and quality [13,14,15,16,17].

Considering, on the one hand, the abundance of bioactive phytochemical compounds in seabuckthorn fruits [3,8,9] and, on the other hand, the minimal demand of this plant in relation to environmental conditions [18], it is interesting to evaluate the biostimulation potential of aqueous extract of seabuckthorn fruits (AESF) in plant cells, using the *Allium cepa* species (onion) as a test plant, a species widely used in cytological determinations [19,20,21].

Animal cell cultures require a complex culture medium, the stability of which is very difficult to maintain during long-term experiments. The likelihood of the interaction between the test substances and the components of the culture medium increases with the increasing complexity of the environment. All of these drawbacks include complicated procedures, long-term experiments, and relatively high costs. However, these shortcomings can be avoided if plant-based materials are used, such as whole plants, seeds, organs, tissues or just cells. One of the most widely used tests in this category is the *Allium* genotoxicity test (or simply the *Allium* test), which is based, in particular, on microscopic observations of abnormalities during mitosis and cytokinesis and subsequent effects on chromosomes in the area of the root division in plants of the genus *Allium*.

The selection of *A. cepa* species as a biological material in the present study was based on the fact that it shows very clear mitotic phases, it has a stable number of chromosomes (2n = 16) with morphological diversity, as well as a clear and rapid response to genotoxic substances.

The aim of this study was to evaluate the biostimulation potential of the aqueous extract of seabuckthorn fruits in *A. cepa* cells. The effects were monitored both at the macroscopic and microscopic level.

## 2. Results

### 2.1. Determination of the pH Value and of the Content of Dry Matter for the Aqueous Extract of Seabuckthorn Fruits (AESF) Variants

**Figure 1:**
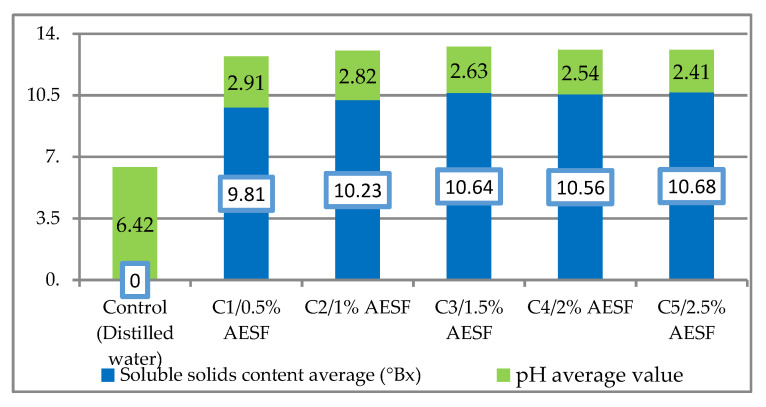
Average content of dry matter (°Brix) and average pH value for the aqueous extract of seabuckthorn fruits (AESF) variants.

Figure 1 shows the pH value and the content in dry matter for each variant of AESF, as average values, following three checks at pH-meter, refractometer, respectively. The pH values ranged within the limits of 2.41 (C5/2.5% AESF) and 2.91 (C1/0.5% AESF), the control version with distilled water having a pH of 6.42. In terms of soluble content, the results ranged from 9.81 ºBrix (C1/0.5% AESF) to 10.68 ºBrix (C5/2.5% AESF).

### 2.2. Evaluation of the Biostimulation Potential of AESF on Germination for A. cepa

**Figure 2:**
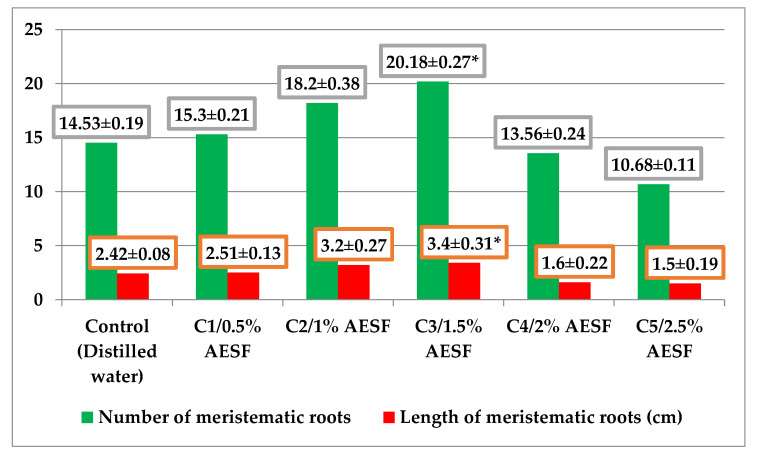
Graphical representation of the macroscopic effects induced by different concentrations of AESF for the *A. cepa* species (average values ± standard error of mean-SEM). * significant at *p* ≤ 0.05, compared to the control (LSD test at a probability level of 0.05% subsequent to ANOVA analysis).

The macroscopic effects of AESF on the *A. cepa* species were evaluated by quantifying the number and length of meristematic roots (Figure 2). The effects identified were both stimulation and inhibition of the two characteristics, depending on the concentration of the AESF. AESF triggered a positive effect on the total number of meristematic roots in the group of variants C1–C3, in which the registered values were 15.30, 18.20, and 20.18, i.e., an increase of 5.29–38.88% compared to the control. On the other hand, at concentrations of 2% and 2.5% of AESF, there was an effect of inhibiting the number of roots, namely 13.56 (C4) and 10.68 (C5), which means a decrease of 6.67–26.49% compared to the control. This trend was also maintained with regard to the length of the meristematic roots, when at AESF concentrations of 0.5–1.5% the effect was to stimulate the growth of the meristematic roots (2.51–3.4 cm), which means 3.71–40.49% longer roots compared to the control. At the same time, at concentrations of 2 and 2.5%, AESF induced the reduction of meristematic growth (1.6 and 1.5 cm, respectively), which means smaller root sizes by 33.88–38.01% compared to the control.

### 2.3. Microscopic Effects of AESF for Meristematic Cells of A. cepa

**Figure 3:**
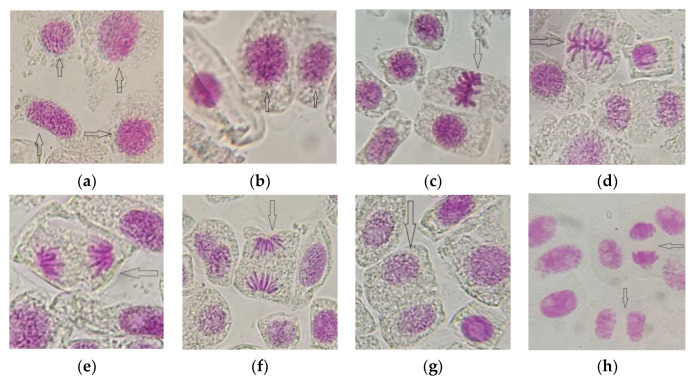
Aspects of mitotic activity on meristematic cells of *A. cepa* exposed to AESF treatment at 0.5 and 1.5% (1000× magnification): cells in prophase (**a**,**b**); cells in metaphase (**c**,**d**); cells in anaphase (**e**,**f**); cells in telophase (**g**,**h**).

The results regarding the influence of AESF at different concentrations on the onion cell activity are illustrated in Table 1. It was found that in the concentration range of 0.5–1.5%, the value of Mitotic index (MI) increased progressively, from 21.52% (C1) to 26.35% (C2) and 31.24 (C3), respectively, compared to the control variant which registered 17.12%. This means an increase of the mitotic activity by 25.70–82.47% for the variants exposed to the action of the AESF against the untreated control, in the concentration ranges mentioned above. The intense mitotic activity resulted in a high frequency of cells in the prophase, metaphase, anaphase, and telophase (Figure 3). On the other hand, in the concentration range 2–2.5% AESF, the value of MI decreased from 15.54% (C4) to 11.27% (C5), which means the depression of mitotic activity decreased by 9.22–34.17% compared to the untreated control.

**Figure 4:**
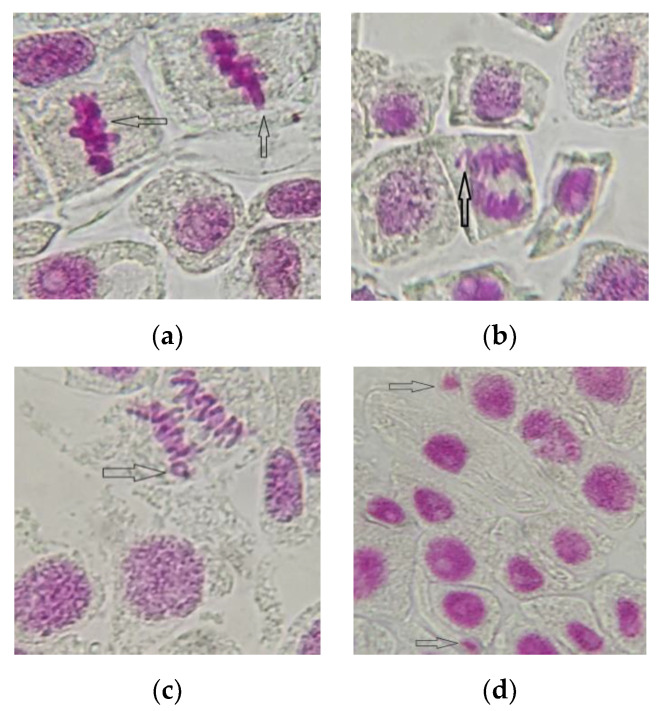
The main chromosomal and nuclear abnormalities identified in meristematic cells of *A. cepa* exposed to the AESF treatment at 2 and 2.5% (1000× magnification): sticky metaphase (**a**), laggard chromosome in anaphase (**b**); ring chromosomes (**c**); micronucleus (**d**).

Exposure to different concentrations of AESF also triggered the appearance of chromosomal and nuclear abnormalities in onion meristematic cells (Figure 4), a significantly higher frequency being recorded at variants C4 (6.51% FCA) and C5 (8.83% FCA). The main anomalies identified were chromosomes of the stickiness type (0.01–1.46%), laggards (0.02–1.25%), and rings (0.01–1.11%), as well as micronuclei (3.06–5.14%), the last ones being observed only in the variants exposed to 2 and 2.5% AESF, respectively.

## 3. Discussion

The ecosystems offer many plants with multiple benefits both for the health of the human body, and for the food, pharmaceutical industry, etc. However, higher plants can be used to obtain valuable products for application in modern and sustainable horticulture and agriculture. The biostimulation activity on crop plants (e.g., the impact on seed germination, plant establishment, growth, and development) is related to the hormonal activity [22,23,24]. The use of botanical extracts will affect the functional plant nutrition associated with increased food quality parameters [25,26]. The results obtained by Godlewska et al. (2020) show a statistically significant influence of some botanical extracts (seabuckthorn, horsetail, hypericum, pea, red clover, etc.) on the growth, composition, and antioxidant activity of cabbage seedlings [26].

In principle, each plant is of interest due to a number of bioactive principles. For example, the aqueous infusion of *Pimpinella anisum* L. seeds has a cytoprotective potential against chemically stimulated wounds in vivo tests [27]; coriander seeds contain a wide array of health beneficial compounds such as minerals, phenolics, fatty acids and essential oils [28]. Extraction conditions and their approach strongly impact on the bioactivity of the achieved infusions from different part of the plants [29].

Due to its multiple uses in the food industry [3], medicine [30,31], cosmetics [32], and the restoration of degraded land in the agricultural ecosystem [11,12], seabuckthorn is commonly known as the “wonder plant” [2], “Holy fruit of Himalayas” [33], or “Virgin Mary’s remedy beans” [34]. Although all the component parts of the plant are valuable, the fruit is mostly used, being the richest in bioactive phytochemical compounds [3,4,35]. This is the reason why this study aimed to evaluate the biostimulation potential of AESF in plant cells, using the *A. cepa* species as test plant. The *Allium* test is recognized as one of the simplest and safest tests for monitoring cell activity under the influence of various chemicals [36].

The pH values determined for each of the five AESF variants ranged within 2.41–2.91, which is correlated with a strong antioxidant effect of the juice obtained from the seabuckthorn fruits, as confirmed by other authors [32,37]. Regarding the content in soluble solids content, the results ranged from 9.81–10.68 °Brix, unlike Gotea et al. (2010), in which the recorded average value was of 11.21 °Brix in the juice wholly extracted from seabuckthorn fruits rather than in the aqueous extract version [38].

Successful germination and seedling development are crucial steps in the growth of a new plant and can be considered as a determinant for plant productivity [39]. High temperatures accelerate the germination process but decrease the activity of some enzymes [40]. On the other hand, some scientists claim that the higher the protein content of the seeds, the faster the germination process [41,42]. The presence of bioactive substances, macro and micronutrients in the chemical composition of various species of spontaneous flora can have positive effects on the growth and development of the root system for plants [43,44]. From this point of view, many researchers highlight the abundance of such useful substances of the seabuckthorn fruits or in other anatomical parts of it [2,4,10,45]. The results reported by Pallavee and Ashwani (2017) show that the juice extracted from seabuckthorn fruits contains water-soluble and fat-soluble vitamins, carotenoids, proanthocyanic flavonoids, pectins, ascorbic acid, amino acids, etc. [46]. Sea buckthorn seed and pulp oils are considered the most valuable components of berries, comprising a unique fatty acid composition, fat-soluble vitamins, and plant sterols [5,8,9,35]. Extensive research has been conducted on the state-of-the-art methods of plant oil extraction to evaluate their efficiency. For example, among these emergent technologies, microwave assisted extraction (MAE) has revealed many advantages such as convenience, shorter processing times and high efficiency [47]. Additionally, oil extraction with ultrasound assistance is considered an improved approach to plant-based products, particularly to the extraction of compounds with lower molecular weight [48]. Ultrasound assisted extraction (UAE) was used by Godlewska et al. (2020) for the production of environmentally friendly and rich in sensitive bioactive compounds [26].

Vitamins play an important role in the growth and development of plants because they are organic compounds that participate in the anabolic and catabolic processes of plants, forming numerous oxidative-reducing systems through which the cellular redox potential is regulated; Vitamins also have the role of enzyme activators in plant cells. Research also suggest the involvement of allelopathy [49] or alkaloids from various extracts [50,51] on the growth and sustainable development of agricultural plants. Similar results have also been reported regarding the beneficial effect of macro and micronutrients from seaweed extract on the growth and development of vegetables, fruits, or other crops [52,53,54].

Exposure of onion bulbs to AESF can either stimulate or inhibit germination, depending on concentration. Although seabuckthorn fruits are considered to be true nutrient deposits [55], the results suggest the effect of stimulating germination in *A. cepa* at AESF concentrations only in the range of 0.5–1.5%. Above these values, the effect is one of germination inhibition. Some studies mention the effect of germination inhibition on *A. cepa* exposed to the action of aqueous extracts from medicinal plants (*Azadirachta indica*, *Morinda lucida*, *Cymbopogon citratus*, *Mangifera indica*, and *Carica papaya*) at concentrations of 1, 5, 10, 25, and 50% [56].

In the present study, AESF at a concentration ≤1.5% induced an effect of stimulating the mitotic activity on *A. cepa*, whereas at a concentration ≥2%, the effect was mitodepressive. The presence of nuclear abnormalities such as MN suggests the genotoxic potential of AESF at ≥2% for onion plant cells. The results reported by other researchers indicate the mitodepressive effect, i.e., of reducing the frequency of MN for human cells [57] and mice cells [18,58] under the action of juice extracted from seabuckthorn fruits at different concentrations. However, data regarding to the cytotoxicity and genotoxicity of the seabuckthorn and its extracts are still scarce. Efforts have been spent to explore the pharmacological activities while only a few studies have focused on the safety evaluation of the plant extracts [59,60]. In a study on mice, the maximum tolerated dose of seabuckthorn oil was >20 mL/kg for mice in relation to acute toxicity, and the no-observed-adverse-effect level was of 10 mL/kg body weight in both male and female rats regarding the 90-day toxicity study [61].

It is likely that little use of chemical fertilizers in agriculture can affect plant productivity [62]. However, the economic losses can be compensated in the long term by increasing the sustainability of the agricultural ecosystem and, implicitly, increasing the quality of life. From this point of view, seabuckthorn can have all the qualities of a basic ecological resource of the future: a low cost resource with excellent ecological plasticity, which resists extreme temperatures (−43 °C to +45 °C), and other unfavorable environmental conditions, such as drought or soil salinity [63]; a natural resource that could be exploited in agriculture as an organic fertilizer for plant growth or remediation of poor soils in nutrients [64]. In order to achieve a sustainable development of the agricultural ecosystem, environmental protection must become an integral part of the development process.

## 4. Materials and Methods

### 4.1. Obtaining the Aqueous Extract from Seabuckthorn Fruits (AESF)

The sea buckthorn fruits were harvested in the first decade of October 2019 from a spontaneous flora in the Gorj region (Romania), where plants are found in their natural habitat. The fresh fruits were washed under running tap water and transformed into a liquid mixture by squeezing them in a fruit juicer. This was followed by the separation of the pulp juice by filtration with the help of a gauze cloth from a dense fabric, placed in a glass funnel.

Five AESF-based test solutions were used, consisting of filtered seabuckthorn juice and distilled water, in the following concentrations: 0.5% (C1), 1% (C2), 1.5% (C3), 2% (C4), and 2.5% (C5), by mixing 0.5, 1, 1.5, 2, and 2.5 mL of juice with 99.5, 99, 98.5, 98, and, respectively, 97.5 mL of distilled water. The control variant (distilled water) was noted C0.

The pH for each AESF variant as well as the dry matter content (°Brix) was determined using the ATC digital pH-meter and the Optika manual refractometer, respectively.

### 4.2. Obtaining the Biological Material

The onion bulbs purchased from the market, of approximately equal size and with no trace of disease or pests, were germinated in glasses, with the germinated disc immersed in the test solutions, for 72 h. Five experimental variants were carried out, corresponding to the concentrations of the test solutions, together with an untreated control, which was germinated in distilled water. The test solution was refreshed every day. All variants were executed in three repetitions.

At the end of the exposure period, the onion bulbs were removed from the test solutions, for macroscopic determinations, establishing the number and length of meristematic roots (cm) while also comparing them with the control variant.

### 4.3. Processing of the Biological Material for the Cytogenetic Study

For the microscopic determinations, the biological material consisting of meristematic roots was excised from the onion bulbs and passed into a fixative solution of ethyl alcohol and acetic acid (3:1 ratio) in the refrigerator for 24 h. The hydrolysis process that followed was carried out in two stages: maintaining the biological material in 1N HCl for 5 min, followed by its maintaining in pure HCl solution with distilled water (1:1 ratio) for 16 min. The coloring of the biological material was achieved by adding 2–3 mL of Schiff reagent to the meristematic roots in glass vials.

Six microscopic slides were executed for each variant, 500 cells per slide were analyzed, at the LCD optical microscope, 1000× magnification power.

The cellular activity was monitored by quantifying the mitotic index and cellular abnormalities. The mitotic index (MI %) was calculated as the total number of cells divided by the total number of cells observed [36]. The frequency of cellular abnormalities (FCA %) was calculated based on the number of aberrant cells related to the total number of cells in the division [36].

### 4.4. Statistical Analysis

Statistical analysis was carried out using MS Excel 2007. The analysis of variance (ANOVA) was used to assess the significant differences between the control variant and each treatment. The differences between treatment means were compared using the Least Significant Difference (LSD) test at a probability level of 0.05% subsequent to the ANOVA analysis [65].

## 5. Conclusions

Seabuckthorn is a plant rich in bioactive compounds and it represents a local resource that grows in spontaneous flora but which can also be grown in various areas, having a high ecological plasticity.

The results of this study indicate the positive effect of aqueous extract from seabuckthorn fruits on the stimulation of germination and vegetative growth on *A. cepa*, by increasing the mitotic activity when used in concentrations of 0.5–1.5%. This suggests the potential of practical use of this extract as a biofertilizer in agriculture, as an alternative to soil contamination with chemical fertilizers and to the progressive degradation of ecosystems. However, further research is needed to confirm this observation. Additionally, in the future it will be necessary to conduct detailed studies of the chemical composition of AESF that can induce the best biostimulatory properties to *A. cepa*.

Use of plant biostimulants can enhance yields and the raw material quality in a sustainable way, with no risks for both the environment and consumers.

## Figures and Tables

**Table 1:** Microscopic effects of AESF at different concentrations on meristematic cells of *A. cepa.*

Concentrations	MI% (Mean ± SEM)	Chromosomal and Nuclear Abnormalities				FCA% (Mean ± SEM)
		S%	L%	R%	MN%	
Ct	17.12 ± 0.93	0.01	0.02	0.01	0.00	0.04 ± 0.11
C1/0.5%	21.52 ± 0.12	0.11	0.06	0.02	0.00	0.19 ± 0.28
C2/1%	26.35 ± 0.23 *	0.10	0.04	0.01	0.00	0.15 ± 0.15
C3/1.5%	31.24 ± 0.21 *	0.02	0.65	0.04	0.00	0.71 ± 0.39
C4/2%	15.54 ± 0.19	1.09	1.25	1.11	3.06	6.51 ± 0.78 *
C5/2.5%	11.27 ± 0.38	1.46	1.21	1.02	5.14	8.83 ± 0.23 *

MI = mitotic index; SEM = standard error of mean; S = stickiness; L = laggards; R = rings; MN = micronuclei; FCA = frequency of cellular abnormalities; * significant at *p* ≤ 0.05, compared to the control (LSD test at a probability level of 0.05% subsequent to ANOVA analysis).
